# Expansion of T Cells with Interleukin-21 for Adoptive Immunotherapy of Murine Mammary Carcinoma

**DOI:** 10.3390/ijms18020270

**Published:** 2017-01-29

**Authors:** Christine K. Zoon, Wen Wan, Laura Graham, Harry D. Bear

**Affiliations:** 1 Department of Surgery, Virginia Commonwealth University Health System, Richmond, VA 23298, USA; 2 Department of Biostatistics, Virginia Commonwealth University School of Medicine, Richmond, VA 23298, USA; wwan@vcu.edu; 3 Division of Surgical Oncology, Virginia Commonwealth University Massey Cancer Center, Richmond, VA 23298, USA; LGraham2@MCVH-VCU.EDU (L.G.); hdbear@vcu.edu (H.D.B.)

**Keywords:** adoptive immunotherapy, mammary carcinoma, IL-21, T lymphocytes

## Abstract

We previously demonstrated that culturing antigen-sensitized draining lymph node (DLN) lymphocytes from BALB/c mice in interleukin (IL)-7/15 after activation with bryostatin/ionomycin (B/I) is superior to culture in IL-2 for expansion, differentiation to cluster of differentiation (CD)8+ cells and anti-tumor activity. We sought to determine whether the substitution or addition of IL-21 to culture had a similar effect. DLN lymphocytes were antigen-sensitized with 4T1 mammary carcinoma 10 days prior to harvest, activated with B/I, and expanded in culture for 7 days with either IL-2, IL-21, IL-2/21, IL-7/15, or IL-7/15/21. Cellular expansion, phenotype, interferon (IFN)-γ responses, and in vivo anti-tumor activity were compared. We found that T cells grown in IL7/15/21 demonstrated significantly greater lymphocyte expansion than IL-2, IL-21, IL-2/21, and IL-7/15 (38.4-fold vs. 5.5, 6.6, 9.5, and 23.9-fold, respectively). Of these expanded cells, IL-7/15/21 significantly expanded the greatest percentage of CD8+ cells (67.1% vs. 22.2%, 47.2%, 47.4%, and 55.3%, respectively), and the greatest number of T central memory cells (T_CM_) compared to IL-2, IL-21 and IL-2/21 (45.8% vs. 11.1%, 7.7%, and 12.1%, respectively). IL-21 and IL-2/21-expanded T cells preferentially differentiated into T naïve cells (T_N_) vs. those expanded in IL-2, IL-7/15 and IL-7/15/21 (27.6% and 23.2% vs. 1.7%, 4.5%, and 10.4%, respectively), and demonstrated the highest IFN-γ levels in vitro. In vivo adoptive immunotherapy (AIT) experiments demonstrated anti-tumor efficacy was equally effective using IL-2, IL-21, IL-2/21, IL-7/15 and IL-7/15/21-cultured lymphocytes vs. control or cyclophosphamide alone, even at lower doses or with greater initial size of tumor prior to treatment.

## 1. Introduction

Adoptive immunotherapy (AIT), infusing ex vivo-expanded lymphocytes for the treatment of cancer in animals and humans, has been a rapidly growing area of interest [1,2,3,4,5]. While demonstrating promising results in murine tumor models, a regimen for human therapy that optimizes both lymphocyte expansion as well as tumor regression remains elusive. AIT enhances ex vivo activation and expansion of T cells outside of the suppressive in vivo tumor environment and allows the immune cells to be “re-programmed” in order to optimize their function. This also allows for additional treatment of the host, including host lymphocyte depletion, prior to re-introducing the selected cells, which decreases immunosuppression and optimizes trafficking and/or proliferation of the infused cells.

In a 4T1 murine mammary cancer model which is weakly immunogenic and aggressively metastatic, we have shown that T cells from tumor antigen-sensitized draining lymph nodes (DLN) can be expanded to significantly greater numbers than the current standard approach using interleukin (IL)-2 alone after exposure to IL-7 and 15, following activation with bryostatin and ionomycin (B/I) [6]. These T cells were able to induce tumor regression as effectively as, if not better than, T cells grown in IL-2 [6]. When bryostatin-1, a macrocyclic lactone derived from a marine invertebrate *Bulgula neritina*, is combined in culture with lymphocytes, it activates protein kinase C, while ionomycin increases intracellular calcium [7,8,9]. When used together, these mimic signaling via the CD3/T cell receptor (TcR) complex, promoting activation and proliferation of T cells [6,10,11,12,13,14,15,16]. B/I selectively activates CD62L^−^ T cells, the “sensitized” T cells capable of anti-tumor activity. Compared to anti-CD3 activation, this effect is unique to B/I activation [7,15]. Recently, we demonstrated that B/I activation in combination with alternate cytokines re-programs human T cells and natural killer (NK) cells, causing them to become resistant to suppression by myeloid-derived suppressor cells [11]. It has been shown in multiple studies that IL-2-expansion induces regulatory T cells and T cell activation-induced cell death, whereas IL-7/15 has been shown by this lab and others to support preferential differentiation of CD8+ T cells towards a central memory (T_CM_) phenotype [6,10,17]. It has been suggested that this central memory phenotype is more effective at inducing tumor regression than terminally differentiated effector cells, which are more likely to be selectively expanded with exposure to IL-2 [17,18]. Encouraged by our results with IL-7/15, we endeavored to add or substitute IL-21, a recently-identified member of the family of cytokines that shares the common γ chain cytokine receptor with IL-2, IL-7 and IL-15, to our expansion regimen [19,20]. Other groups have previously demonstrated the potent immunomodulatory effects on T cells of IL-21 [21,22,23,24]. The resultant T cell phenotype stimulated by exposure to IL-21 has varied, with some studies demonstrating an increase in T_CM_ cells, while other studies claimed inhibition [19,25,26]. Therefore, we also performed flow cytometry analysis of cells after exposure to B/I and expansion in various cytokines to elucidate which phenotypes were preferentially selected. Finally, it has been suggested that both IL-15 and IL-21 are able to enhance the in vivo anti-tumor effects of CD8+ T cells and, in some cases, potentiate tumor regression [24,27,28,29,30,31,32]. We endeavored to discover if antigen-sensitized T cells cultured in B/I with IL-21 exposure alone or combination with IL-2 or IL-7/15 would increase their expansion. We also aimed to study the effect on T cell phenotype and interferon γ production in vitro, and whether it would increase in vivo anti-tumor activity.

## 2. Results

### 2.1. Expansion Comparison of 4T1 Draining Lymph Node (DLN) Cells in Interleukin (IL)-2, IL-21, IL-2/21, IL-7/15 or IL-7/15/21

**Figure 1:**
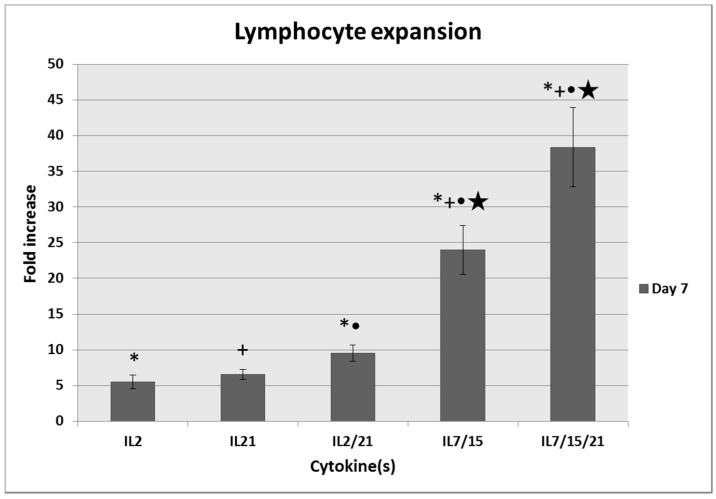
Fold increase of T cells expanded for 7 days in either interleukin (IL)-2, IL-21, IL-2/21, IL-7/15 or IL-7/15/21 after being pulsed with bryostatin/ionomycin (B/I) and IL-2. * IL-2 vs. IL-2/21, IL-7/15, and IL-7/15/21 (*p* ≤ 0.008); **+** IL-21 vs. IL-7/15, and IL-7/15/21 (*p* < 0.0001); ● IL-2/21 vs. IL-7/15 and IL-7/15/21 (*p* < 0.0001); ★ IL-7/15 vs. IL-7/15/21 (*p* = 0.028).

Averaged over seven repeated experiments, cells cultured in IL-7/15 and IL-7/15/21 after activation with B/I expanded to significantly higher numbers than those cultured in with IL-2, IL-21 or IL-2/21 (23.9 and 38.4-fold increase vs. 5.5, 6.6 and 9.5-fold respectively; *p* < 0.008; Figure 1). In addition, expansion of cells cultured in IL-7/15/21 was significantly greater than for those cultured in IL-7/15 (*p* = 0.028). We also noted that when we continued to culture cells for 14 days, the fold increases in IL-7/15 and IL-7/15/21-cultured cells continued to increase, whereas the number of cells cultured in IL-2, IL-21 and IL-2/21 remained static. This finding is similar to our earlier studies demonstrating that not only do IL-2 cultured cells not continue to expand beyond 7 days in culture, but cells begin to decline in number and have poor viability after that time.

### 2.2. T Cell Phenotype

**Figure 2:**
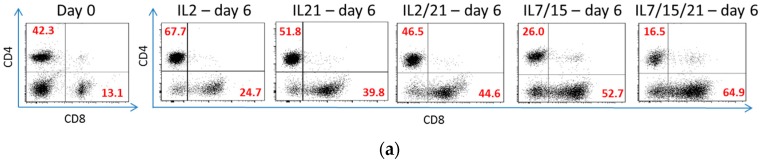

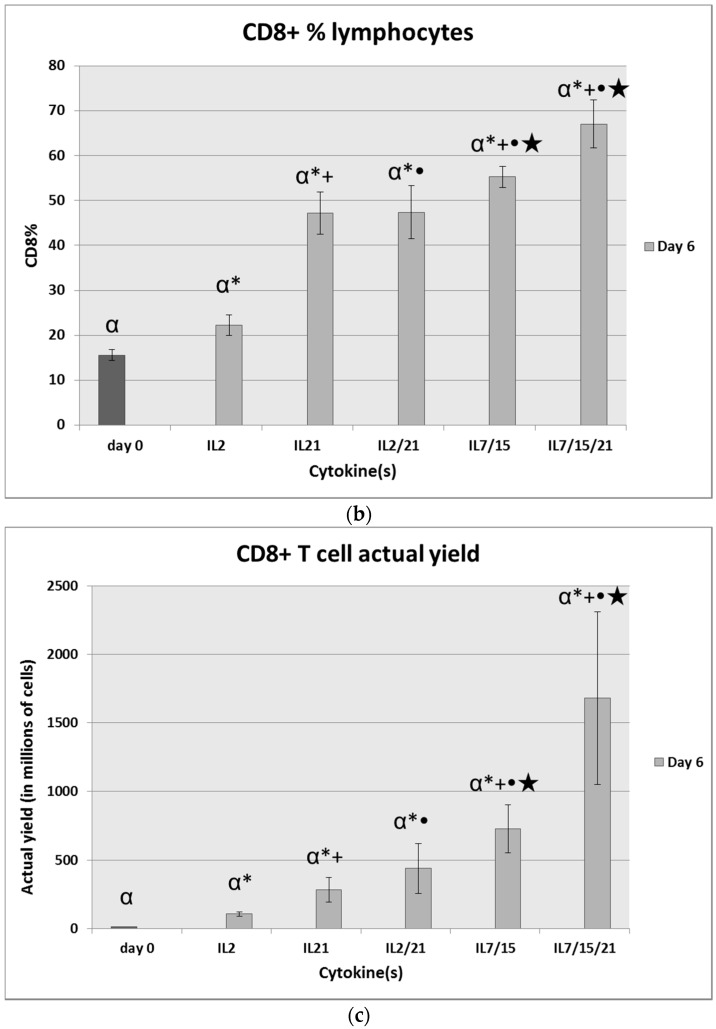
(**a**) Representative flow cytometry experiment of T cell lymphocytes stained with cluster of differentiation (CD)4 or CD8 on day 0 before B/I pulse vs. after B/I pulse and 6 days in culture with IL-2, IL-21, IL-2/21, IL-7/15 or IL-7/15/21. Cells were forward scatter, side scatter (FSC/SSC) gated on viable lymphocytes, and the percentages of cells that were CD4+ and CD8+ were determined (numbers in red); (**b**) Average percentages of CD8+ T cells expanded over five experiments before (day 0) and after (day 6) exposure to B/I and 6 days of IL-2, IL-21, IL-2/21, IL-7/15 or IL-7/15/21. **α** day 0 vs. all day 6 (*p* ≤ 0.0002); * day 6 IL-2 vs. all other day 6 (*p* < 0.0001); **+** day 6 IL-21 vs. day 6 IL-7/15 and day 6 IL-7/15/21 (*p* = 0.02 and < 0.0001, respectively); ● day 6 IL-2/21 vs. day 6 IL-7/15 and day 6 IL-7/15/21 (*p* = 0.02 and < 0.0001, respectively); and ★ day 6 IL-7/15 vs. IL-7/15/21 (*p* = 0.005); (**c**) The percentages of CD8+ lymphocytes were multiplied by the number of total cells expanded to obtain actual yield of cells in that subset. **α** day 0 vs. all day 6 (*p* < 0.0001); * day 6 IL-2 vs. all other day 6 (*p* ≤ 0.03); **+** day 6 IL-21 vs. day 6 IL-7/15 and day 6 IL-7/15/21 (*p* = 0.0002 and < 0.0001, respectively); ● day 6 IL-2/21 vs. day 6 IL-7/15 and day 6 IL-7/15/21 (*p* = 0.003 and < 0.0001, respectively); and ★ day 6 IL-7/15 vs. IL-7/15/21 (*p* = 0.0005).

After 6 days in culture, flow cytometry was used to analyze the phenotypes of the lymphocytes expanded in different cytokines. As shown in Figure 2a, a representative experiment of five total experiments was performed. CD4 and CD8 sorting was performed on viable lymphocytes on day 0 prior to B/I and IL2 activation and after 6 days in culture with the various cytokines. Over these five experiments, IL-7/5/21-cultured lymphocytes were noted to have the highest percentage of CD8+ T cell lymphocytes, with an average percentage of 67.1% compared to day 0 (15.6% *p* < 0.0001), IL-2 (22.2% *p* < 0.0001), IL-21 (47.2% *p* < 0.0001), IL-2/21 (47.4% *p* < 0.0001), and IL-7/15 (55.3% *p* = 0.005; Figure 2b). In fact, IL-21, IL-2/21 and IL-7/15, in addition to IL-7/15/21, expanded with a significantly higher proportion of CD8+ T lymphocytes compared to IL-2 (all *p* < 0.0001). The only two groups that were not statistically different from each other were IL-21 and IL-2/21. When total cell count after expansion and phenotypic proportion were taken into account, lymphocytes exposed to IL-7/15/21 produced 1681.5 million CD8+ T cells on day 6 versus 726.1 million (*p* = 0.0005) for cells grown in IL-7/15, 437.3 million cells grown in IL-2/21 (*p* < 0.0001), 282.8 million cells grown in IL-21 (*p* < 0.0001), and 104.7 million cells grown in IL-2 over 6 days (*p* < 0.0001) from a starting population that averaged 13 million cells on day 0 for each group (Figure 2c). Again, the only groups not statistically significant from each other were IL-21 and IL-2/21.

**Figure 3:**
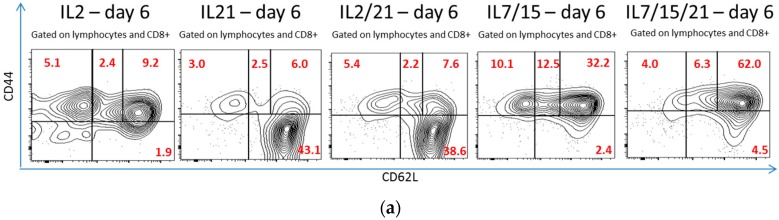

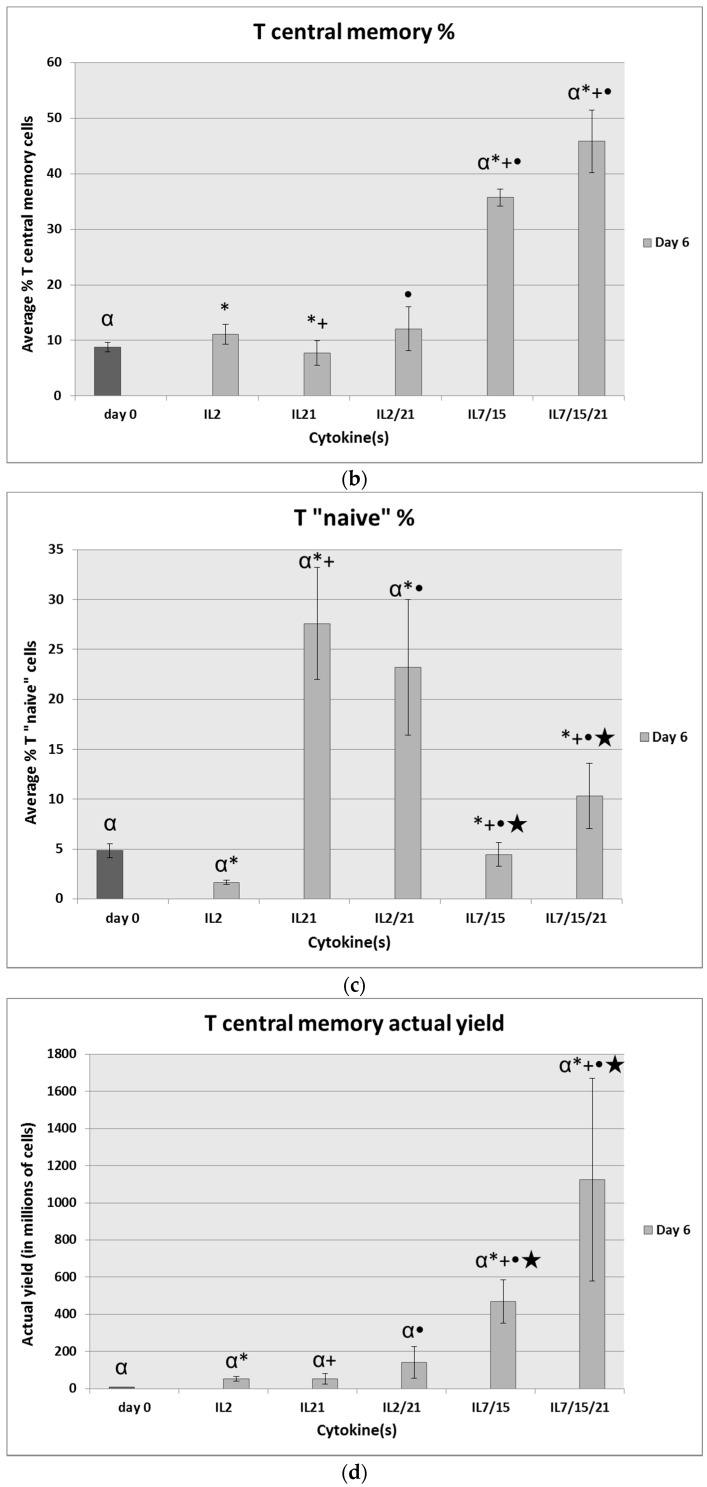

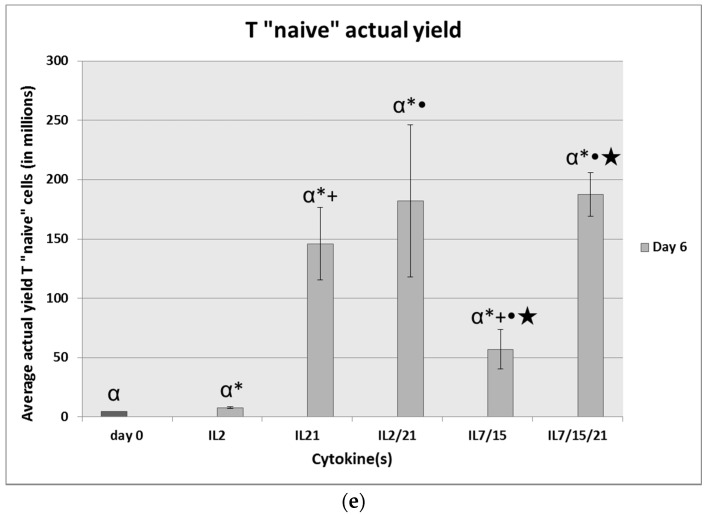
(**a**) Representative flow cytometry experiment of CD8+ T cell lymphocytes stained with CD44 and CD62L after B/I pulse and 6 days in culture with IL-2, IL-21, IL-2/21, IL-7/15 or IL-7/15/21. Cells were forward scatter/side scatter (FSC/SSC) gated on viable lymphocytes, and the percentages of cells that were T effector (CD44+CD2L−), T effector memory (CD44+CD62Llo), T central memory (CD44+CD62Lhigh) and T ”naïve” (CD44−CD62L+) were determined (numbers in red); (**b**) Average percentages of CD8+ T central memory phenotype T cells expanded over five experiments before (day 0) and after (day 6) exposure to B/I and 6 days of IL-2, IL-21, IL-2/21, IL-7/15 or IL-7/15/21. **α** day 0 vs. day 6 IL-7/15 and IL-7/15/21 (*p* < 0.0001); * day 6 IL-2 vs. day 6 IL-21, IL-7/15 and IL-7/15/21 (*p* = 0.05, *p* = 0.0005, *p* < 0.0001, respectively); **+** day 6 IL-21 vs. day 6 IL-7/15 and day 6 IL-7/15/21 (*p* < 0.0001, *p* < 0.0001, respectively); and ● day 6 IL-2/21 vs. day 6 IL-7/15 and day 6 IL-7/15/21 (*p* < 0.0001, *p* < 0.0001, respectively); (**c**) Average percentages of CD8+ T “naïve” phenotype T cells expanded over five experiments before (day 0) and after (day 6) exposure to B/I and 6 days of IL-2, IL-21, IL-2/21, IL-7/15 or IL-7/15/21. **α** day 0 vs. day 6 IL-2, IL-21 and IL-2/21 (*p =* 0.04, *p* = 0.001 and *p* = 0.004, respectively); * day 6 IL-2 vs. all other day 6 (*p* < 0.0001, *p* < 0.0001, *p* = 0.05, *p* = 0.0015); **+** day 6 IL-21 vs. day 6 IL-7/15 and day 6 IL-7/15/21 (*p =* 0.0002, *p* = 0.01, respectively); ● day 6 IL-2/21 vs. day 6 IL-7/15 and day 6 IL-7/15/21 (*p* = 0.0007, *p* = 0.06, respectively); and ★ day 6 IL-7/15 vs. IL-7/15/21 (*p* = 0.05); (**d**) The percentages of CD8+ T central memory (T_CM_) lymphocytes were multiplied by the number of total cells expanded to obtain actual yield of cells in that subset. **α** day 0 vs. all day 6 (*p* ≤ 0.001); * day 6 IL-2 vs. day 6 IL-7/15 and IL-7/15/21 (*p* = 0.0005, *p* < 0.0001, respectively); **+** day 6 IL-21 vs. day 6 IL-7/15 and day 6 IL-7/15/21 (*p* < 0.0001); ● day 6 IL-2/21 vs. day 6 IL-7/15 and day 6 IL-7/15/21 (*p* = 0.0003 and < 0.0001, respectively); and ★ day 6 IL-7/15 vs. IL-7/15/21 (*p* = 0.03); (**e**) The percentages of CD8+ T “naïve” (T_N_) lymphocytes were multiplied by the number of total cells expanded to obtain actual yield of cells in that subset. **α** day 0 vs. all day 6 (*p* ≤ 0.02), * day 6 IL-2 vs. all other day 6 (*p* ≤ 0.001); **+** day 6 IL-21 vs. day 6 IL-7/15 (*p* = 0.01); ● day 6 IL-2/21 vs. day 6 IL-7/15 (*p* = 0.007); and ★ day 6 IL-7/15 vs. IL-7/15/21 (*p* = 0.0009).

The expanded cells were also analyzed for their CD8+ T cell subsets on day 6 and compared to phenotypic expression on day 0 and among the different groups. A representative experiment is demonstrated in Figure 3a, demonstrating the T cell phenotypes of CD8+ T cells after B/I activation and expansion in various cytokines for 6 days. When sorted for T central memory (T_CM_) phenotype (CD44+, CD62Lhi), IL-7/15/21-cultured cells had the highest percentage of this phenotype (45.8%) compared to day 0 (8.8% *p* < 0.0001), IL-2-cultured cells (11.1% *p* < 0.0001), IL-21-cultured cells (7.7% *p* < 0.0001), and IL-2/21-cultured cells (12.1% *p* < 0.0001), as shown in Figure 3b. There was no significant difference between IL-715/21-cultured T cells and IL-7/15-cultured cells (35.7% *p* = 0.2) for this phenotype. IL-21 and IL-2/21-expanded T cells, however, expanded the highest percentages of CD44−, CD62L+ cells, a so so-called T “naïve” (T_N_) population as it has been described in the literature [1,33]. IL-21 and IL-2/21 expanded 27.6% and 23.2% of T “naïve” cells vs. day 0 (4.8% *p* ≤ 0.004), IL-2 (1.7% *p* < 0.0001), IL-7/15 (4.5% *p* ≤ 0.0007) and IL-7/15/21 (10.4% *p* = 0.01 and *p* = 0.06, respectively), as shown in Figure 3c. Again, when total cell count after expansion and phenotypic proportion were taken into account, not surprisingly, lymphocytes expanded in IL-7/15/21 had the highest yield of T_CM_ cells averaged over five experiments, with 1124.1 million cells vs. 468.8 million for IL-7/15-expanded cells (*p* = 0.03), 140.5 million for IL-2/21-expanded cells (*p* = 0.0001), 53.3 million for IL-21-expanded cells (*p* < 0.0001), and 54.2 million for IL-2-expanded cells (*p* < 0.0001) from a starting amount of 7.3 million cells on day 0 (*p* < 0.0001), as shown in Figure 3d. For the subset of T_N_ cells, IL-7/15/21-expansion and IL-2/21-expansion resulted in the highest yield values averaged over five experiments, with 187.6 million and 182.1 million T_N_ cells, respectively (Figure 3e). In the case of IL-7/15/21-expansion this was due to the overall higher number of total cells expanded vs. IL-2/21-expansion where the high yield of T_N_ cells was due to the high percentage of that particular phenotype which was expanded despite lower total post-expansion cell numbers. IL-21 expanded the next highest total T_N_ cell yield with 145.9 million, which was not statistically significantly different to IL-7/15/21 and IL-2/21. IL-7/15-expansion led to a T_N_ cell yield of 57 million, which was significantly more than for day 0 or IL-2 (*p* < 0.0001 and *p* = 0.001 respectively), and also significantly less than IL-7/15/21, IL-2/21 and IL-21 yields (*p* = 0.0009, *p* = 0.007, and *p* = 0.01). IL-2-expanded cells yielded an average of 7.7 million T_N_ cells, which was only slightly higher, yet still significantly more than the day 0 population at 4.5 million (*p* = 0.02).

### 2.3. Interferon (IFN)-γ ELISA

**Figure 4:**
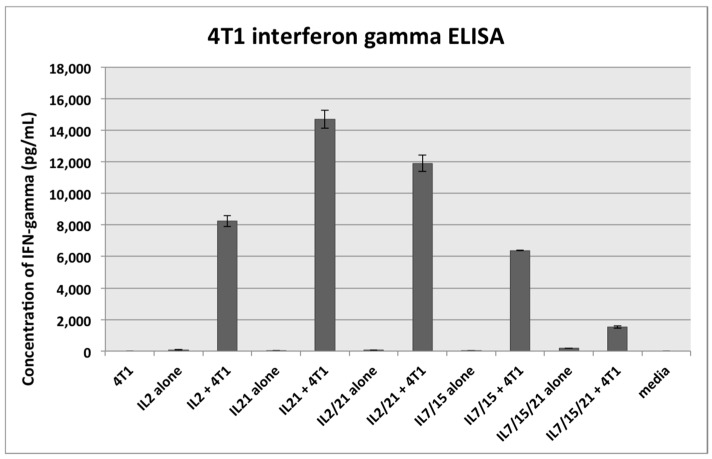
Interferon-γ ELISA assay. B/I pulsed and IL-2, IL-21, IL-2/21, IL-7/15 and IL-7/15/21-exposed draining lymph node (DLN) lymphocytes were co-cultured with irradiated 4T1 cells or media alone for 24 h. 4T1 irradiated cells alone were also used as a control. IL-2-expanded lymphocytes co-cultured with 4T1 vs. all other groups (*p* ≤ 0.02), IL-21-expanded/co-cultured lymphocytes (*p* ≤ 0.006), IL-2/21-expanded/co-cultured lymphocytes (*p* ≤ 0.006), IL-7/15-expanded/co-cultured lymphocytes (*p* ≤ 0.02), and IL-7/15/21-expanded/co-cultured lymphocytes (*p* < 0.0001).

DLN T cells activated with B/I and cultured with IL-2, IL-21, IL-2/21, IL-7/15 or IL-7/15/21 for 7 days were harvested and cultured for 24 h alone or with irradiated 4T1 murine mammary carcinoma cells to stimulate IFN-γ release. As shown in Figure 4, IL-21 and IL-2/21-expanded T cells co-cultured with irradiated 4T1 resulted in the largest release of IFN-γ at 14,700 pg/mL and 11,900 pg/mL respectively, which were statistically different from each other (*p* = 0.006). These were far higher than all the other co-cultured groups, with all *p* values < 0.0001. IL-2-co-cultured T cells produced 8240 pg/mL of IFN-γ, IL-7/15-co-cultured cells produced 6380 pg/mL, and IL-7/15/21-co-cultured cells produced the least of the co-cultured groups at 1524 pg/mL. Due to the small standard deviations these were all statistically significantly different from each other with the highest *p* value = 0.02 between IL-2 and IL-7/15-co-cultured cells. Controls in the form of 4T1 irradiated cells alone, IL-2, IL-21, IL-2/21, IL-7/15 and IL-7/15/21-cultured cells alone (without 4T1 in co-culture) and media alone were used. The amount of IFN-γ produced by controls was insignificant compared to the co-culture groups. Of note, similar to our previous research with IL-2 and IL-7/15-expanded cells co-cultured with irradiated 4T1 tumor cells, we found that IL-7/15-cultured cells produced less IFN-γ than did the IL-2-cultured T cells. As shown previously as well, the IFN-γ secretion in response to 4T1 cells by DLN cells activated with B/I and expanded in vitro is tumor antigen-specific [16,34].

### 2.4. Anti-Tumor Efficacy

**Figure 5:**
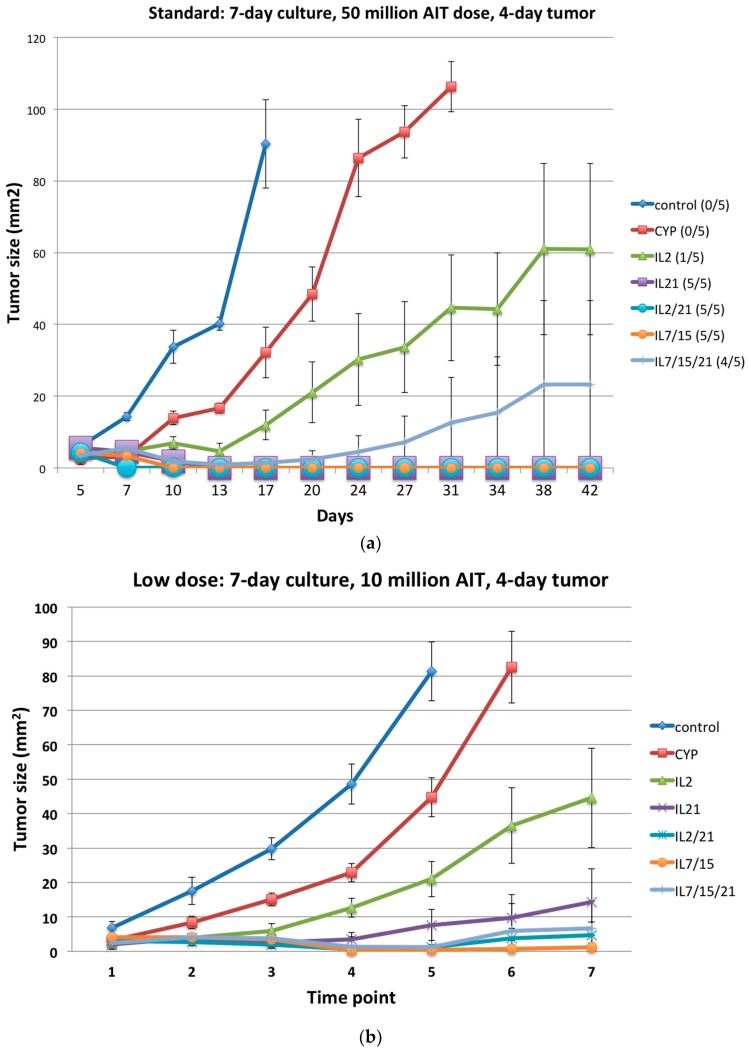

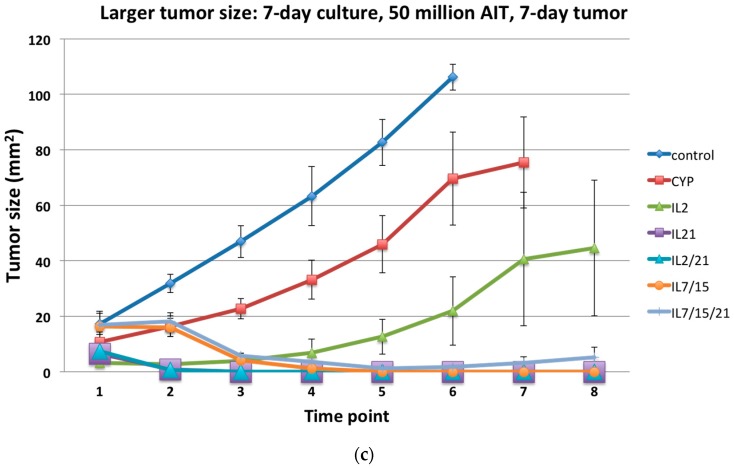
(**a**) Adoptive immunotherapy (AIT) against 4T1 subcutaneous tumors with T cells activated with B/I and cultures in IL-2, IL-21, IL-2/21, IL-7/15, IL-7/15/21. Recipient BALB/c mice were injected in the flank with 10^4^ 4T1 cells and randomized into seven treatment groups: untreated control, cyclophosphamide (CYP)-only (100 mg/kg), and CYP + AIT with B/I activated DLN cells from 4T1-sensitized BALB/c mice cultured in IL-2, IL-21, IL-2/21, IL-7/15 or IL-7/15/21 (50 × 10^6^ cells/mouse). CYP was given 3 days after tumor inoculation and cultured lymphocytes were infused intravenously (IV) the following day (day 4). Mean tumor sizes +/− standard error are charted over time (in days). Numbers in parentheses to the right indicate the number of mice with complete tumor regression (cure) per total number of mice in each group; (**b**) Adoptive immunotherapy (AIT) against 4T1 subcutaneous tumors with T cells activated with B/I and cultures in IL-2, IL-21, IL-2/21, IL-7/15, IL-7/15/21 at a lower dose (10 million cells/mouse), averaged over three experiments. Recipient BALB/c mice were injected in the flank with 10^4^ 4T1 cells and randomized into seven treatment groups: untreated control, cyclophosphamide (CYP)-only (100 mg/kg), and CYP + AIT with B/I activated DLN cells from 4T1-sensitized BALB/c mice cultured in IL-2, IL-21, IL-2/21, IL-7/15 or IL-7/15/21 (10 × 10^6^ cells/mouse). CYP was given 3 days after tumor inoculation and cultured lymphocytes were infused IV the following day (day 4). Mean tumor sizes +/− standard error are charted over time (in time points which represent a 2–3 day time period); (**c**) Adoptive immunotherapy (AIT) against 4T1 subcutaneous tumors with T cells activated with B/I and cultures in IL-2, IL-21, IL-2/21, IL-7/15, IL-7/15/21 with initial larger tumor (7 day tumor) averaged over two experiments. Recipient BALB/c mice were injected in the flank with 10^4^ 4T1 cells and randomized into seven treatment groups: untreated control, cyclophosphamide (CYP)-only (100 mg/kg), and CYP + AIT with B/I activated DLN cells from 4T1-sensitized BALB/c mice cultured in IL-2, IL-21, IL-2/21, IL-7/15 or IL-7/15/21 (10 × 10^6^ cells/mouse). CYP was given 6 days after tumor inoculation and cultured lymphocytes were infused IV the following day (day 7). Mean tumor sizes +/− standard error are charted over time (in time points which represent a 2–3 day time period).

Mice treated with cyclophosphamide alone or cyclophosphamide plus AIT with the standard dose of lymphocytes (50 million cells/mouse intravenously) expanded for 7 days in IL-2, IL-21, IL-2/21, IL-7/15, IL-7/15/21 after 4T1 flank injections (4-day tumor growth) demonstrated significantly better flank tumor shrinkage compared to the control group (Figure 5a; *p* = 0.02, *p* < 0.0001, *p* < 0.0001, *p* < 0.0001, *p* < 0.0001, *p* < 0.0001, respectively). In addition, the IL-2, IL-21, IL-2/21, IL-7/15 and IL-7/15/21-cultured DLN cells were significantly better at shrinking tumors than cyclophosphamide alone (*p* = 0.02, *p* < 0.0001, *p* < 0.0001, *p* < 0.0001, *p* < 0.0001, respectively). Of note, only 1 of the 5 mice in the IL-2 group was cured of tumor (no visible tumor 42 days after injection), whereas in the IL/15/21 group, four of the five mice were cured and all five mice were cured of tumor in the IL-21, IL-2/21 and IL-7/15 groups. Even at a decreased dose of lymphocytes (10 million cells/mouse) the IL-2, IL-21, IL-2/21, IL-7/15 and IL-7/15/21 groups were significantly better at slowing or decreasing tumor growth than the control or the cyclophosphamide-alone groups (Figure 5b; all *p* < 0.0001). Likewise, when the flank tumor was allowed to grow for 7 days rather than 4 days prior to AIT treatment, the IL-2, IL-21, IL-2/21, IL-7/15 and IL-7/15/21 groups all had significant slowing of tumor growth or tumor regression when compared to the control or cyclophosphamide-alone groups (Figure 5c; *p* < 0.03).

## 3. Discussion

As we have demonstrated previously in the B16 melanoma system, we have now demonstrated in mammary carcinoma that culturing B/I-activated lymphocytes from tumor-sensitized mice in IL-7/15/21 results in a much higher yield of viable cells than those grown in IL-2, IL-21, IL-2/21 and even IL-7/15. A recent review highlighted factors that were associated with effective adoptive immunotherapy [1]. In this review, the magnitude of tumor regression was strongly correlated with the absolute number of infused cells. In addition, the least differentiated cells (T_CM_ cells) demonstrated the most effective anti-tumor activity. We demonstrate that the expansion of lymphocytes in IL-7/15/21 following activation with B/I results in a dramatic increase in the total number of cells, with a final actual yield of CD8+ T cells over one billion from an average starting number of 13 million on day 0. Of these CD8+ T cells, approximately half were T_CM_ cells. Lymphocytes expanded in IL-7/15/21 were then found to be as effective as lymphocytes cultured in other cytokines at impeding tumor growth or curing tumors in vivo without vaccine or exogenous cytokine administration. This was true even at a lower dose of lymphocytes or with larger tumors. This finding suggests that lymphocytes grown in IL-7/15/21 are capable of achieving high numbers of total cells, the majority of which are CD8+ T_CM_ cells that have significant anti-tumor effect against tumors in vivo. In addition, this also demonstrates that by using these cytokine combinations, T_CM_ cells need not be sorted to a pure population to be effective in their anti-tumor function, which for adoptive immunotherapy purposes is more clinically feasible. However, should sorting be desired, the total number of cells produced with expansion renders this goal possible.

We observed consistently that lymphocytes expanded in IL-21 were comparable in their total expansion to IL-2 (current standard). IL 2/21 was significantly better than IL-2 at expansion but not nearly to the extent of those cultured in IL-7/15 or IL-7/15/21. IL-21 and IL-2/21were very similar in the percentage of CD8+ cells expanded, significantly more than IL-2, but less than IL-7/15 and IL-7/15/21. Of these CD8+ cells, however, IL-21 and IL-2/21 expanded the largest number of T “naïve” cells, those that are CD62L+CD44–. In addition, cells cultured in IL-21 or IL-2/21 demonstrated the highest IFN-γ release response when combined with irradiated 4T1 cells. This is in contrast to what we found with the B16 melanoma model, but similar to our recent observations comparing IL-2 and IL-7/15 in the 4T1 mammary carcinoma model [6,10]. Here, we observed a shift away from IFN-γ production for IL-7/15 and IL-7/15/21-expanded lymphocytes vs. IL-2, IL-21 and IL-2/21-expanded lymphocytes. This is thought to be characteristic of differentiation toward T_CM_ cells and different from terminally differentiated effector cells [18,35]. The equally efficacious anti-tumor activity of the IL-7/15 and IL-7/15/21 lymphocytes in vivo, despite lower effector function, is in agreement with findings of other authors who suggest that T cells with greater cytotoxicity or IFN-γ release may be less effective at inducing tumor regression than memory T cells [17,18,30]. In many studies, it has been suggested that exposure to IL-21 alone results in lymphocytes that remain in or differentiate into a minimally differentiated phenotype (CD62L+CD44–), and that these lymphocytes have greater anti-tumor capacity relative to cells expanded in other γ chain cytokines [10,27,36]. Some have described the lymphocytes expanded in IL-21 with this minimally differentiated phenotype as T stem cell memory (T_SCM_)-like cells [33]. It is explained that because of the unique ability of IL-21 to sustain signal transducer and activator of transcription 2 (STAT2) activation, the cells maintained a “TSCM-like state that is associated with high proliferative potential and long-term T cell survival” [33]. In our experiments, although lymphocytes expanded in IL-21 or the combination of IL-2/21 indeed were enriched for the “minimally differentiated” phenotype of CD62L+CD44−, this did not translate into a higher proliferative capacity unless in combination with IL-7 and IL-15. In addition, this phenotype was not significantly better at impeding tumor growth or curing tumors in vivo compared to those cytokines that expanded other T cell phenotypes.

## 4. Methods

### 4.1. Mice

Virus-free BALB/c mice (National Cancer Institute, Bethesda, MD, USA) between 8 and 12 weeks of age, caged in groups of five or fewer, were provided with food and water ad libitum. All guidelines of the Virginia Commonwealth University Institutional Animal Care and Use Committee, which conform to the American Association for Accreditation of Laboratory Animal Care and the U.S. Department of Agriculture recommendations for the care and humane experimental use of animals, were followed.

### 4.2. Tumor Cell Lines

4T1 mammary tumor cell line was kindly provided by Jane Tsai of the Michigan Cancer Foundation, Detroit, Michigan. Cells were maintained in Dulbecco’s Modified Eagle Medium (DMEM) with 10% heat-inactivated fetal calf serum (Hyclone, Logan, UT, USA), 1 mM sodium pyruvate, 100 U/mL penicillin, and 100 µg/mL streptomycin (Sigma, St. Louis, MO, USA; modified DMEM). Tumor cells were harvested for inoculation of mice with 0.05% trypsin–EDTA (Fisher, Pittsburgh, PA, USA).

### 4.3. Draining Lymph Node Sensitization

Donor mice were vaccinated in the footpad with 5 × 10^5^ 4T1 cells. Ten days after the vaccination, popliteal tumor draining lymph nodes (tDLNs) were harvested under sterile conditions and placed in Roswell Park Memorial Institute (RPMI) media per our previous protocol [6].

### 4.4. Lymphocyte Activation and In Vitro Expansion

After harvest, DLN were dispersed into a single cell suspension at 1 × 10^6^ cells/mL and activated by incubation with 5 nM bryostatin-1 (provided by the National Cancer Institute, Bethesda, MD, USA) and 10 nM ionomycin (Calbiochem, San Diego, CA, USA), B/I, and 80 U/mL of rIL-2 (Chiron, Emeryville, CA, USA) as per our previous protocol [6]. Cells were washed and resuspended at 1∓2 × 10^6^ cells/mL with 40 U/mL of recombinant interleukin (rIL)-2, IL-21, IL-2 + IL-21, IL-7 + IL-15 or IL-7 + IL-15 + IL-21 (10 ng/mL each). The cells were allowed to proliferate in culture for an additional 6 days and were split every 2–3 days to maintain 1–2 × 10^6^ cells/mL concentration. Fresh cytokines were added at each split to achieve the same final concentration as at culture initiation. Samples were assessed for the number of viable cells by trypan blue exclusion at various days of culture.

### 4.5. Adoptive Immunotherapy

Host mice were inoculated in the left flank with 1 × 10^4^ 4T1 cells, which were left to grow for either 4 days (standard time interval) or 7 days (elongated time interval). One day prior to AIT, mice were pretreated with cyclophosphamide (CYP, Mead Johnson, Princeton, NJ, USA), 100 mg/kg intraperitoneally (IP). After 6 days in culture, the B/I activated and expanded DLN lymphocytes were washed twice in phosphate buffered saline, filtered through a 70-µm nylon mesh strainer (Invitrogen, Carlsbad, CA, USA), and 50 million (regular dose) or 10 million (low dose) cells were injected intravenously (IV) in 0.5 mL into host mice. No systemic cytokines or vaccinations were administered to these tumor-bearing mice.

### 4.6. Flow Cytometry

T cells isolated from DLN were stained with a panel of antibodies on day 0 (immediately after B/I activation) and on day 6 after activation in vitro. These stained cells were analyzed by multicolor flow cytometry for surface markers on a FACSAria Canto flow cytometer. Fluorescently labeled antibodies directed against the following markers were obtained from Biolegend and eBiosciences: CD4, CD8, CD62L, and CD44. Appropriate isotype controls were used in all cases. T cell subsets analyzed were T effector (T_E_) CD44+CD62L−, T effector memory (T_EM_) CD44+CD62Llow, T central memory (T_CM_) CD44+CD62Lhigh and “T naïve” (“T_N_”) CD44−CD62L+, as described previously [37,38,39].

### 4.7. IFN-γ Release Assay

Interferon-γ (IFN-γ) release in supernatants from tumor-sensitized and B/I-activated and expanded lymphocytes (for 7 days), in response to stimulation with irradiated 4T1 for 24 h, was assayed using BD OptEIA mouse IFN-γ ELISA sets from BD Biosciences (San Jose, CA, USA).

### 4.8. Tumor Measurements

In all the AIT experiments, tumor growth was monitored with biweekly measurements of perpendicular diameters and animals were euthanized by CO_2_ inhalation when tumor area was greater than 100 mm^2^ or if a mouse appeared ill [6]. Complete tumor regression was defined as the absence of a measurable tumor for three consecutive measurements.

### 4.9. Statistical Analysis

At least five mice were included in each experiment. Each outcome was summarized with basic descriptive statistics such as mean and standard deviation for each treatment group. Repeated measures analyses of variance through linear mixed models were used to compare the various cytokine conditions in fold increase cell numbers on day 7 from baseline in lymphocytes, each T cell phenotype (including both percentage and total number of cells of CD4 and CD8, and T_E_, T_EM_, T_CM_, and T_N_), in ELISA, and in tumor size. Pairwise comparisons of the various cytokines were made and tested at type I error of 5% by a *t*-test with the random measurement errors estimated by the linear mixed model. Normality was checked for each analysis. Natural logarithm was used in the data of fold increase of T cell expansion, and percentage and total numbers of T cell phenotypes, and square root was used in ELISA for normality. SAS 9.4 was used for all analyses.

## 5. Conclusions

We demonstrate here that lymphocytes expanded in the triple combination of IL-7/15/21 have a large proliferative capacity, significantly greater than the current standard of IL-2 expansion, and are capable of producing billions of not only CD8+ T cells but also CD8+ T_CM_ cells from a starting population of tens of millions of lymphocytes. These lymphocytes are as effective as IL-2-expanded cells at slowing tumor growth or curing tumors, even at low doses of cells or with large starting tumors. This occurs without the need to sort the cells for specific T cell phenotypes, nor is exogenous cytokine administration required, which avoids potential for severe side effects. Vaccinations in vivo also were not required. These results demonstrate great promise for producing large numbers of highly efficacious anti-tumor lymphocytes for use in adoptive immunotherapy in future human trials.
